# Ultrafast terahertz magnetometry

**DOI:** 10.1038/s41467-020-17935-6

**Published:** 2020-08-25

**Authors:** Wentao Zhang, Pablo Maldonado, Zuanming Jin, Tom S. Seifert, Jacek Arabski, Guy Schmerber, Eric Beaurepaire, Mischa Bonn, Tobias Kampfrath, Peter M. Oppeneer, Dmitry Turchinovich

**Affiliations:** 1 Fakultät für Physik, Universität Bielefeld, Universitätsstr. 25, 33615 Bielefeld, Germany; 2 Max Planck Institute for Polymer Research, Ackermannweg 10, 55128 Mainz, Germany; 3 Department of Physics and Astronomy, Uppsala University, Box 516, 75120 Uppsala, Sweden; 4 Terahertz Technology Innovation Research Institute, University of Shanghai for Science and Technology, JunGong Road 516, 200093 Shanghai, China; 5 Department of Materials, ETH Zurich, Hönggerbergring 64, 8093 Zurich, Switzerland; 6 Université de Strasbourg, CNRS, Institut de Physique et Chimie des Matériaux de Strasbourg (UMR 7504), 23 rue du Loess, BP 43, 67034 Strasbourg Cedex 2, France; 7 Fachbereich Physik, Freie Universität Berlin, Arnimallee 14, 14195 Berlin, Germany

**Keywords:** Terahertz optics, Ultrafast photonics, Magnetic properties and materials, Spintronics

## Abstract

A material’s magnetic state and its dynamics are of great fundamental research interest and are also at the core of a wide plethora of modern technologies. However, reliable access to magnetization dynamics in materials and devices on the technologically relevant ultrafast timescale, and under realistic device-operation conditions, remains a challenge. Here, we demonstrate a method of ultrafast terahertz (THz) magnetometry, which gives direct access to the (sub-)picosecond magnetization dynamics even in encapsulated materials or devices in a contact-free fashion, in a fully calibrated manner, and under ambient conditions. As a showcase for this powerful method, we measure the ultrafast magnetization dynamics in a laser-excited encapsulated iron film. Our measurements reveal and disentangle distinct contributions originating from (i) incoherent hot-magnon-driven magnetization quenching and (ii) coherent acoustically-driven modulation of the exchange interaction in iron, paving the way to technologies utilizing ultrafast heat-free control of magnetism. High sensitivity and relative ease of experimental arrangement highlight the promise of ultrafast THz magnetometry for both fundamental studies and the technological applications of magnetism.

## Abstract (web summary)

External stimuli can induce significant changes in the magnetisation of a material; however, these changes can occur very rapidly, making measurements difficult. Herein the authors demonstrate a method of ultrafast magnetometry, enabling the detection of the rapid magnetization changes.

## Introduction

Magnetism^1^ has traditionally been a major topic of fundamental research. It also underpins numerous modern technologies ranging from high-capacity memories^2,3^ to novel concepts in computing and data transfer^4,5^. Since the discovery of sub-picosecond spin momentum removal in a magnetic system two decades ago^6^, the field of ultrafast magnetism has become a rapidly growing area in modern condensed matter physics (see e.g. refs. ^7–9^ and references therein). We owe our present understanding of ultrafast magnetization dynamics to a range of advanced laser-based experimental methods, all of which, however, feature certain drawbacks in terms of limited precision, ambiguity of measurement interpretation, or limited applicability under realistic device conditions. Spin-resolved surface science methods such as time-resolved photoemission^10,11^ require ultra-high vacuum conditions, pristine sample surfaces, and technically challenging detection schemes. These constraints preclude the measurements on encapsulated materials or devices, as well as experimentation under typical device working conditions. Optical probes such as the magneto-optical Kerr effect (MOKE)^12,13^ or optical second-harmonic generation^14^ are versatile but are limited to the optical probe penetration depth.

Further, the interpretation of MOKE signals on sub-picosecond timescales is not straightforward: pump-induced change in the magnetooptical constant of a material can make a significant contribution to the MOKE signal itself, which currently remains the subject of intense research^12,13^.

Over a decade ago, THz electromagnetic emission from optically excited magnetic metallic structures^15,16^ was reported for the first time, and was assigned to the presence^16^ or temporal modification^15^ of magnetization in laser-excited samples. A number of studies followed (see, e.g. refs. ^17–24^), reporting on the THz emission from laser- or THz-excited metallic and dielectric magnetic structures, and tracing this emission back to ultrafast spin- and electron-dynamics of various origins.

THz emission spectroscopy relies on a fundamental principle of electrodynamics: a time-varying magnetization *M*(*t*) or polarization *P*(*t*) in the sample acts as a source of electromagnetic radiation, known as magnetic *E*_M_(*t*) or electric *E*_P_(*t*) dipole emission, respectively (see “Methods”). When the temporal variation of *M*(*t*) or *P*(*t*) occurs on the ultrafast, (sub-)picosecond timescale, the resulting radiation contains THz frequencies. The THz electromagnetic waves, in turn, experience considerably weaker absorption in metals, semiconductors, and dielectrics in comparison to ultraviolet, visible, and infrared lightwaves. Such a high penetration power becomes a natural advantage of the THz waves, making them especially suitable for probing relatively thick, multilayer structures and devices (see, e.g. ref. ^25^), where optical probes can fail. Unlike other methods, THz emission spectroscopy can be easily implemented under ambient conditions, and can be applied to encapsulated samples or devices. Importantly, such a contact-free measurement reveals the ultrafast magnetization dynamics in the sample as a whole, and not only within the probed surface region. Most crucially, however, the principles of THz emission spectroscopy must allow for direct and fully calibrated determination of the quantity of fundamental interest, the ultrafast magnetization dynamics in the sample *M*(*t*), from the measurable quantity—magnetic dipole emission *E*_M_(*t*). It must be emphasized here that the magnetic dipole emission *E*_M_(*t*) is connected to its source, instantaneous magnetic moment *M*(*t*), in the most straightforward way, not involving any potentially time-dependent coupling constants. Nonetheless, such measurements have so far remained a challenge. For example, in a dedicated comparative study^18^ the discrepancy was identified between the timescales of *M*(*t*) estimated via THz emission and MOKE methods. Therefore, in spite of all its promise, the full potential of THz emission spectroscopy for studies of ultrafast magnetism still remains to be shown.

Here, we demonstrate an accurate and practical ultrafast magnetometry method, based on THz emission spectroscopy under ambient conditions. As a proof-of-principle, we rigorously determine the ultrafast magnetization dynamics *M*(*t*) in a laser-excited encapsulated iron film, based on the measured electric field component *E*_M_(*t*) of the laser-driven magnetic dipole THz emission from the sample. This measurement is sufficiently sensitive to clearly resolve and disentangle various contributions to the complex magnetization dynamics in the sample, occurring on sub-picosecond and picosecond timescales. Our measurements are in quantitative agreement with the results of theoretical modeling.

## Results

### Sample and experiment

**Fig. 1:**
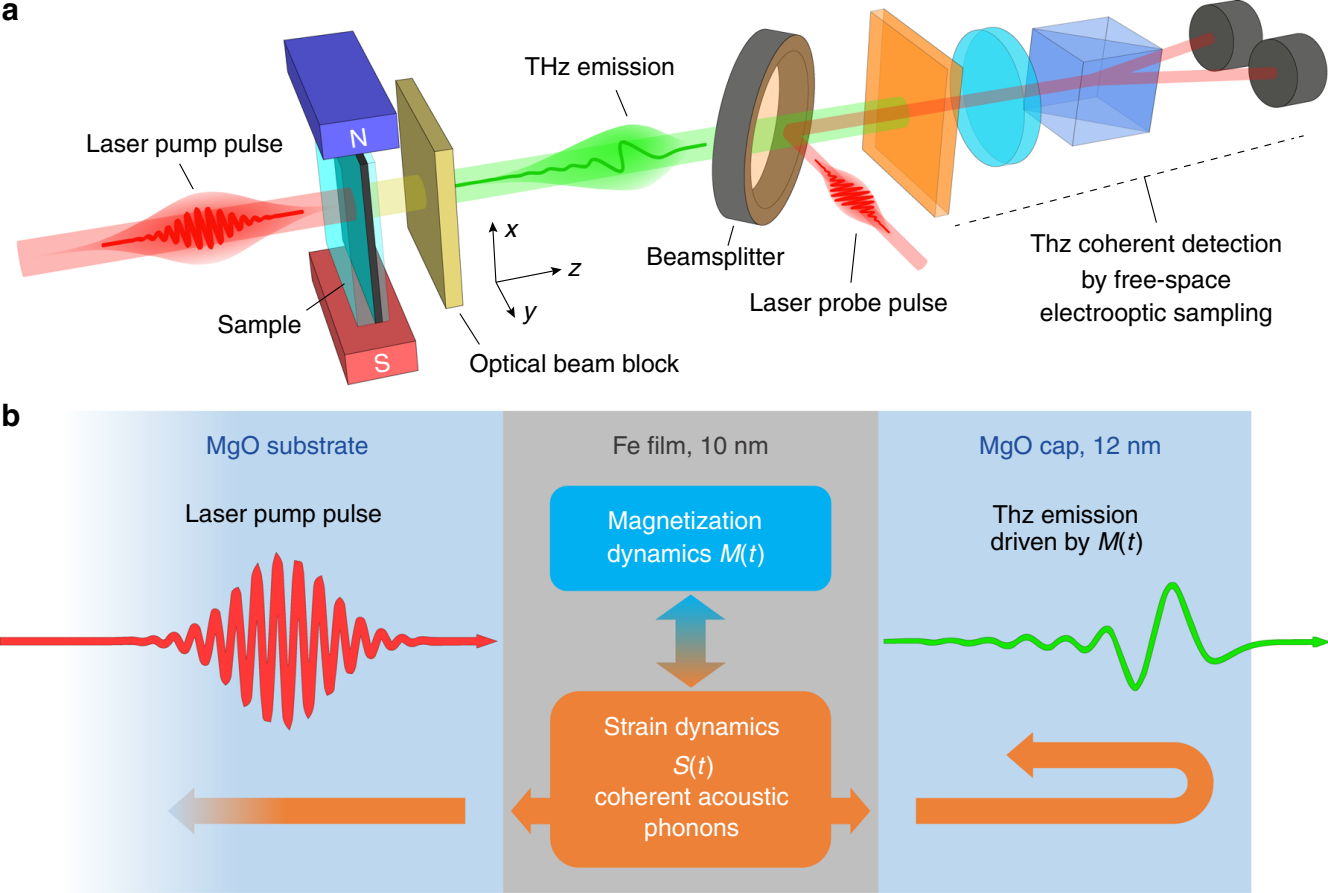
Schematic of the experiment and the sample. **a** Sketch of the THz emission experiment: the sample is placed in the static in-plane magnetic field of 63.4 mT, and excited with a 100 fs, 800 nm laser pulse at normal incidence. The THz emission from the sample in the forward-propagating direction is detected using free-space electro-optic sampling in a 1 mm ZnTe crystal, without any intermediate focusing or additional guiding. **b** Schematic of the sample: 10-nm-thick bcc Fe film, grown on a 0.5-mm-thick MgO substrate, and capped with 12-nm-thick MgO cap. The excitation gradient of the electrons in the Fe film in such a configuration is negligible, and hot-electron diffusion from the Fe film is blocked by MgO substrate and cap. This eliminates the possibility of any ISHE-type mechanism, which relies on the motion of spin-polarized electrons in the sample, in the laser-induced magnetization dynamics *M*(*t*). The only possible contributions to *M*(*t*) in the sample remain hot-electron-driven transient demagnetization causing incoherent hot-magnon excitation (primary dynamics), and modulation of exchange interaction in Fe due to generated coherent acoustic phonons *S*(*t*) propagating in the structure (secondary dynamics).

The schematic of our experiment is shown in Fig. 1a. Our sample was a 10-nm-thick single-crystalline Fe film epitaxially grown on <100> MgO substrate, and capped with a 12-nm-thick MgO layer as shown in Fig. 1b (see “Methods”). The sample was optically excited at normal incidence, using linearly polarized 100 fs transform-limited pulses of 800 nm central wavelength from an amplified Ti:Sapphire laser. The laser pump beam was collimated with the excitation laser spot on the sample of 19.6 mm^2^ (1/e^2^ width), and the corresponding excitation fluence was ranging from 0.25 to 1.02 mJ cm^−2^. The sample was placed in a static in-plane magnetic field of *B* = 63.4 mT, as shown in Fig. 1a. The forward-propagating THz emission from the sample was measured in the far-field at a distance of 10.3 cm from the sample, without any intermediate optical components, using free-space electro-optic sampling (FEOS) in a 1-mm-thick <110> ZnTe crystal gated by 100 fs, 800 nm laser pulses^26^. This detector configuration provided the required sensitivity and acceptance of spectral bandwidth (see “Methods”). A THz-transparent laser beam block was placed behind the sample to stop the propagation of the remainder of the pump laser beam in the setup (see Fig. 1a).

### Challenges in implementation of the method

Before we proceed with the results of our measurements and their interpretation, we outline here the challenges in the successful implementation of ultrafast THz magnetometry. The detailed solutions to these challenges are presented in Methods. First of all, THz magnetic dipole radiation *E*_M_(*t*) due to magnetization dynamics *M*(*t*) inside the sample must be uniquely identified as such, and properly isolated. Yet, under certain conditions, the THz emission from the photoexcited magnetic samples can, in fact, be strongly dominated by electric dipole emission *E*_P_(*t*), driven by a time-varying electric current *J*(*t*) = d*P*/d*t* in the sample (see “Methods”). This emission originates from the ultrafast inverse spin-Hall effect (ISHE)^17,19^, converting the non-radiating laser-driven hot spin currents in the sample into THz-emitting charge currents. While originating from the ultrafast superdiffusion of hot spin-polarized electrons, these THz-emitting conduction currents are in fact an intricate convolution of ultrafast spin currents^27^ with the complex process of natural ultrafast conductive response in a material, involving, e.g., energy-, density-, spin- and time-dependent electron mobility (see, e.g. refs. ^25,28–30^). Therefore, this ISHE-mediated THz emission does not carry direct, but only indirect information about the laser-induced dynamics of the magnetic state in the material *M*(*t*). Yet, as we show below, the ISHE-driven emission easily obscures the much weaker THz emission driven purely by magnetization dynamics *M*(*t*), which remains the quantity of fundamental interest. We overcome this challenge by a proper sample design, where the electronic motion is precluded, thus eliminating the ISHE mechanism; and by performing control measurements, fully supported by the simulations, that unambiguously confirm the magnetic dipole origin of the detected THz fields (see “Methods”). It should be noted here, that all THz emission experiments on metallic magnetic systems known to us^17–21^, are, in fact, dealing with the situation of non-local spin dynamics, and hence with predominantly ISHE-type electric dipole THz emission. This includes the pioneering observation of THz emission from an optically pumped Ni/Cr nanostructure made by one of us, Beaurepaire et al.^15^, which was initially assigned to the process of laser-driven transient demagnetization *M*(*t*) in Ni. Our present understanding^17,19,31^, however, clearly points to the electric dipole nature of this THz emission^15^ via the ISHE mechanism, enabled by the superdiffusion of laser-excited spin-polarized electrons from Ni to Cr, and resulting in the THz-emitting in-plane charge current in the Cr layer. As such, even after more than two decades since the first experiments on ultrafast magnetism^6^, and after more than a decade since the pioneering observations of THz emission from magnetic materials^15,16^, the fundamental process of laser-driven transient demagnetization in ferromagnetic metals has not been identified so far in a clear, unambiguous and calibrated manner, to the best of our knowledge.

Another challenge is a rigorous and fully calibrated reconstruction of the source of magnetic dipole radiation, i.e., the magnetization dynamics in the sample *M*(*t*), from the observable, which is the THz electro-optic signal (EOS) detected in the far-field, many centimeters away from the sample. As the propagation and detection of the THz fields significantly modify their temporal structure and spectral content, such a reconstruction becomes another critical step in the successful implementation of ultrafast THz magnetometry: minor discrepancies in the signal reconstruction will lead to a significant error in the recovered *M*(*t*). We address this challenge by introducing an experimental calibration procedure to a known reference THz emitter, confirmed by a numerical simulation of the THz propagation function in the spectrometer (see “Methods”).

The thickness of the Fe layer used in this work was chosen to be 10 nm, which ensures a virtually uniform pump intensity distribution within the film (see “Methods”). As a result, a gradient of superdiffusive motion of photoexcited electrons in the film is precluded. The MgO substrate and 12-nm-thick MgO cap layer (see Fig. 1b) serve as diffusion barriers, inhibiting the motion of laser-excited electrons out of Fe. As a result, the motion of spin-polarized electrons in our MgO/Fe/MgO sample is precluded, thus eliminating the possibility for ISHE-type mechanisms and leaving the process of pure transient demagnetization *M*(*t*) as the only possible source of THz electromagnetic emission from this sample. As a control sample, we have grown a nominally identical Fe film on an MgO substrate, but now capped with a metallic Pd film of 5 nm thickness. In such a sample, the superdiffusion of laser-excited spin-polarized electrons from Fe to Pd is possible, thus leading to THz emission via the ISHE mechanism^17,19^. The polarization of the electric field component of the measured THz emission from both MgO/Fe/MgO and MgO/Fe/Pd samples was always perpendicular to the direction of the applied magnetic field. Rotating the polarization of the incident laser pulse about the incidence axis had no effect on the THz emission from both samples.

**Fig. 2:**
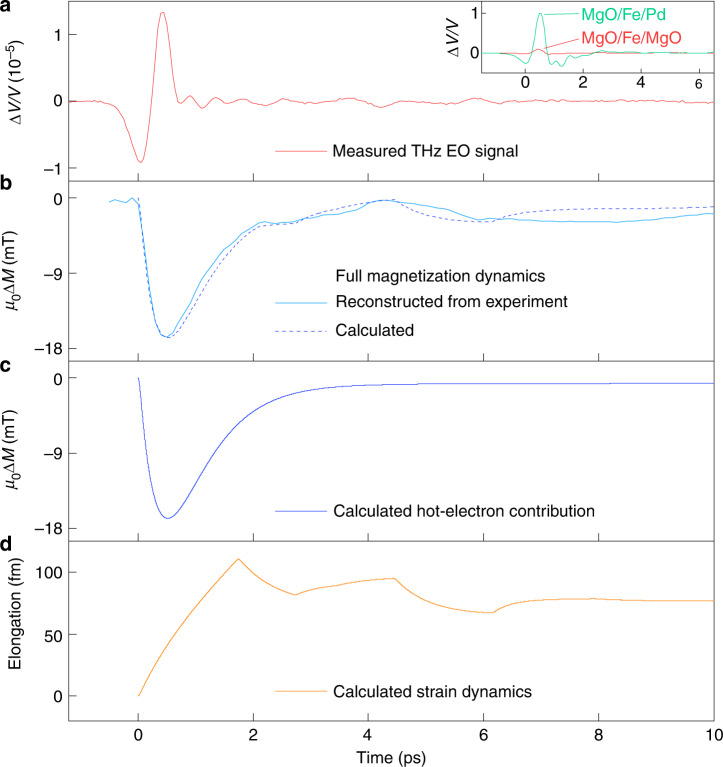
Observable quantities: measured electro-optic signal of the THz emission, the reconstructed magnetization dynamics *M*(*t*), and its theoretical simulation. **a** Measured FEOS signal of the THz emission from MgO/Fe/MgO sample, under the excitation of 0.51 mJ cm^−2^. Inset: comparison of FEOS signal obtained from MgO/Fe/MgO sample via pure magnetic dipole emission due to *M*(*t*), red line; and MgO/Fe/Pd sample, dominated by the electric dipole emission via ISHE, green line. **b** Reconstructed magnetization from experimental EOS (solid line) and simulated magnetization dynamics (dashed line). Simulation conditions are identical to the experiment. *μ*_0_*M*_0_ = 2.15 T—equilibrium magnetization in Fe (see “Methods”). **c** Hot electrons excite hot magnons that drive primary demagnetization dynamics. **d** Dynamics of elongation of the Fe film due to the generated coherent optical phonon pulse. Complete magnetization dynamics *M*(*t*) comprises both incoherent hot-magnon and coherent acoustic phonon contributions.

Exemplary measured THz EOS from the MgO/Fe/MgO and MgO/Fe/Pd samples, obtained under identical excitation conditions, are shown in the inset of Fig. 2a. The THz emission from the Pd-capped sample exceeds that from the MgO-capped one by about a factor of 10 in amplitude. The dominant mechanisms of THz emission from the MgO/Fe/MgO and MgO/Fe/Pd samples can be differentiated, and uniquely identified as transient demagnetization and ISHE, respectively, using the symmetry analysis of the polarity of measured THz signals (see “Methods” for details). The order of magnitude difference in strength between the THz emission from MgO/Fe/MgO and MgO/Fe/Pd samples shown in the inset of Fig. 2a emphasizes the importance of creating the dielectric diffusion barriers in the sample, blocking the superdiffusion of hot spin-polarized electrons, and thereby suppressing the ISHE THz emission mechanism, for the observation of the pure transient demagnetization processes. We note here that any broken inversion symmetry of the film would also lead to the parasitic electric dipole emission, which was, however, observed to be negligible in our samples (see “Methods”).

### Reconstruction of magnetization dynamics from THz emission

Having proven that the THz emission from the MgO-capped Fe nanofilm is exclusively related to the local dynamics of magnetic order in laser-excited Fe, the measured THz EOS can be rigorously reconstructed to its source, the ultrafast magnetization dynamics *M*(*t*) in Fe. Our reconstruction protocol is based on the rigorous experimental and theoretical characterization of the complex propagation function, which connects the THz radiation source, i.e., magnetization dynamics *M*(*t*) in the sample, to the observable, the THz EOS of the magnetic dipole emission *E*_M_(*t*). Once the complex propagation function is established, its deconvolution allows the quantitative extraction of the quantity of interest, magnetization dynamics *M*(*t*). Our reconstruction protocol is described in detail in the “Methods” section.

In Fig. 2a, we show the exemplary as-measured THz EOS, and in Fig. 2b the reconstructed magnetization dynamics *M*(*t*), in our MgO/Fe/MgO sample (excitation fluence of 0.51 mJ cm^−2^, experimental configuration as shown in Fig. 1a). The reconstructed *M*(*t*) signal contains two clearly visible components: (i) a fast transient demagnetization signal with the amplitude on the order of *μ*_0_Δ*M* = 16.6 mT, with an initial drop on the timescale of about 500 fs, and recovery on the timescale of about 1.7 ps; and (ii) a time-delayed contribution of a rather complex shape, with an amplitude of about 2.5 mT, and a total duration of several picoseconds. We attribute the initial fast component to the pure hot-magnon-driven transient demagnetization process in laser-excited Fe: the laser-excited hot electrons in Fe excite nonequilibrium magnons, which in turn (incoherently) drive the demagnetization process. The dynamics of this demagnetization is reasonably well-understood, and has by now been observed by a variety of other experimental methods (see, e.g. refs. ^11,13,32,33^). We assign the second, time-delayed component in the reconstructed magnetization dynamics *M*(*t*) to the magnetoelastic effect driven by the laser-excited coherent acoustic phonon propagation in the MgO/Fe/MgO structure. The mechanism of the magnetoelastic effect is as follows. Subsequent to the laser excitation, a lattice strain is impulsively generated in Fe via electron-phonon coupling^34^, thus producing a coherent acoustic phonon pulse propagating away from the Fe film surface. This acoustic pulse will undergo reflections on the acoustic discontinuities within the structure (see e.g. ref. ^35^): partial reflections on the Fe/MgO interfaces, and total reflection on the outside cap layer MgO/air interface, as shown in Fig. 1b, thus returning back to the Fe film. The propagation of such a multiply reflected coherent acoustic phonon pulse through Fe leads to the transient modification of interatomic distances in Fe, modulating the exchange interaction in the material, and hence leading to an additional, strain-induced demagnetization channel in Fe (see, e.g. refs. ^36–38^ and references therein). We note that, to the best of our knowledge, the direct measurement of such an acoustically driven magnetization dynamics on the ultrafast (sub-)picosecond timescale, and its co-existence with the direct laser-driven demagnetization via hot magnons, has not been reported before.

### Comparison of experiment and theory

Here, we have theoretically modeled the entire magnetization dynamics in the laser-excited MgO/Fe/MgO structure employing a methodology that uses as building blocks a microscopic multi-temperature model (MMTM), lattice strain dynamics and first-principles calculations (see “Methods” for details). In Fig. 2b, we show the exemplary measured and simulated *M*(*t*) signals, corresponding to identical excitation conditions (excitation fluence of 0.51 mJ cm^−2^), and demonstrating very good quantitative agreement. Figure 2c shows the calculation of the isolated hot-magnon driven transient demagnetization dynamics, which occurs on a ~500 fs timescale and recovers on a timescale of ~1.7 ps. In Fig. 2d the calculated dynamics of elongation of laser-excited Fe film is presented, corresponding to the experimental conditions in Fig. 2b: the initial strain in the 10-nm-thick Fe film builds up on a ~2 ps timescale, followed by slow (>100 ps) relaxation, convoluted with the multiple reflections of the strain waves within the Fe film, as well as the return of the strain wave reflected from the outside MgO/air interface of the sample back into the Fe film. The theoretically simulated magnetization dynamics curve *M*(*t*) in Fig. 2b includes the contributions of the hot-magnon driven transient demagnetization (Fig. 2c), and the coherent-phonon-driven magnetoelastic effect (Fig. 2d). Note that the ~2.6 mT amplitude of the acoustically driven demagnetization signal in Fig. 2b is only about 6.5 times smaller than that from the direct laser-driven transient demagnetization of 16.7 mT.

**Fig. 3:**
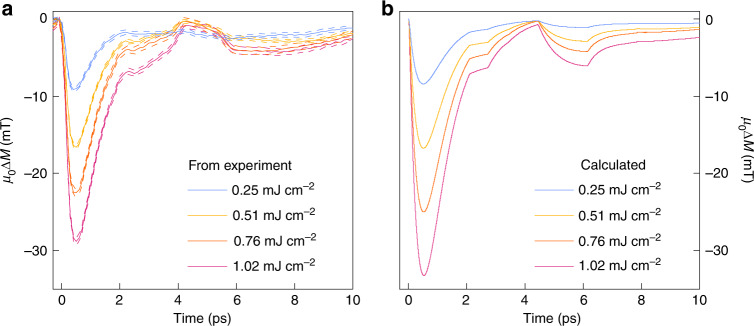
Comparison of measured and simulated magnetization dynamics *M*(*t*) in laser-excited MgO/Fe/MgO sample in this work. **a** Ultrafast magnetization dynamics in the Fe film at various laser pump fluences in the range 0.25–1.02 mJ cm^−2^ with errors shown by dashed lines, and **b** results of simulation corresponding to the experimental scenario in (**a**). The error bars in (**a**) were determined from the standard errors in the measured electro-optic signals (from 200 individual measurements). The experimental error was then propagated in the reconstruction protocol, leading to the intervals as indicated.

In Fig. 3a, b we show the entirety of the measured magnetization dynamics signals *M*(*t*) in this work under the excitation fluence in the range 0.25–1.02 mJ cm^−2^, and the results of the first-principles calculations as described above, fully corresponding to the experimental conditions. The maximum observed hot-magnon driven demagnetization reaches 28.8 mT (corresponding to the change in magnetic moment density of 2.26 × 10^4^ J T^−1^ m^−3^), while the strain-driven demagnetization reaches 3.5 mT (0.18 × 10^4^ J T^−1^ m^−3^) at the strongest excitation. Hence, the maximum measured hot-magnon – and coherent phonon-driven demagnetizations of *μ*_0_Δ*M* = 28.8 mT and *μ*_0_Δ*M* = 3.5 mT, respectively, correspond to about 1.3% and 0.16% of the equilibrium magnetization in Fe of *μ*_0_*M*_0_ = 2.15 T. In this respect, we would like to emphasize the high sensitivity provided by our ultrafast magnetometry method, which allows one to reliably observe the ultrafast magnetization dynamics on the order of 10^−4^ *M*_0_ (see Figs. 2, 3). Generally, the ratio of the amplitudes of these two components of transient demagnetization signal is maintained as $\Delta M_{{\mathrm{hot - magnon}}}{\mathrm{/}}\Delta M_{{\mathrm{phonon}}}\, {\approx} \,7$ for the whole range of excitation fluences used in the experiment. Such a high relative efficiency of ultrafast magnetization control by strain, as compared to direct laser excitation of electrons, is quite remarkable, and points to the promise of efficient ultrafast magnetic technologies controlled by acoustic signals, as opposed to traditional optical, electric, or magnetic stimuli. Notwithstanding, the ratio $\Delta M_{{\mathrm{hot - magnon}}}{\mathrm{/}}\Delta M_{{\mathrm{phonon}}} \,{\approx} \,7$ points to the dominating role of ultrafast magnon excitation for the process of laser-driven femtosecond demagnetization.

Again, the experimental data and the results of modeling are in good agreement: the calculation captures both the temporal features and the magnitudes of the measured transient demagnetization signals. The small discrepancy in the shapes of measured and modeled magnetoelastic contribution might be caused by minor thickness fluctuations in the sample within the laser excitation spot, affecting the local arrival times of the acoustic phonon pulse, and thus leading to a smearing of the overall magnetoelastic signal. Further, the influence of phenomena such as the Elliott-Yafet electron-phonon spin-flip scattering process^39,40^, phonon-magnon coupling^41^ or heat diffusion^42^, which have not been considered in our theoretical modeling, and whose contribution have been estimated only phenomenologically, might also play a certain role. For example, in our theoretical modeling a linear dependence of the electron-magnon coupling parameter (see “Methods”) on the absorbed optical pump fluence was assumed, and its value was established phenomenologically from the experimental data taken at the intermediate pump fluence of 0.51 mJ cm^−2^ (see Fig. 2). The reason for such an assumption is the current lack of solid experimental and theoretical knowledge regarding this important physical parameter. Yet, as can be seen in the comparison of the entirety of our experimental and theoretical data presented in Fig. 3, the maximum calculated hot-magnon driven demagnetization at the maximum pump fluence of 1.02 mJ cm^−2^ exceeds that observed in the experiment by about 25%. While such a discrepancy is not very large, it only emphasizes the need to understand more in-depth the processes governing the magnetic response of materials to ultrafast external stimuli. Our experimental method can significantly contribute to such development.

## Discussion

To summarize, we have presented a rigorous method for ultrafast magnetometry based on THz emission spectroscopy. In our proof-of-principle experiment, we observe the ultrafast demagnetization dynamics in laser-excited Fe, and uniquely identify and disentangle two contributions to this process: (i) a hot-electron-driven emission of incoherent hot magnons leading to ultrafast transient demagnetization, and (ii) a coherent acoustic-phonon-driven magnetoelastic effect. Our observations are fully supported by the results of theoretical modeling. To the best of our knowledge, this is the first simultaneous observation of both hot-magnon and coherent-phonon-driven ultrafast magnetization dynamics in a magnetic system, with hot magnons being the dominating effect. Our observations demonstrate the potential of our highly sensitive method for both fundamental research and industrial applications of magnetism. The magnetization dynamics driven by coherent acoustic phonon propagation in iron corresponds to the elongation of the Fe film on the order of only tens of fm (see Fig. 2). It is even more so remarkable, that the effects related to such tiny elongations could be clearly revealed in the experiment using THz radiation, i.e., sub-mm electromagnetic waves having 10 orders of magnitude longer wavelength. The surprisingly strong magnetoelastic contribution to ultrafast magnetization dynamics, leading to a clearly measurable electromagnetic emission in the THz range, holds promise for spintronic technologies based on a purely acoustic and, where possible, heat-free ultrafast control of magnetism.

## Methods

### Pump intensity distribution within the iron film

The refractive index of iron at the pump wavelength around 800 nm is *n* = 2.94 + 3.39*i*^43^, yielding the optical penetration depth on the order of 20 nm under bulk conditions (Beer-Lambert Law). However, multiple interferences of the pump light in the film result in a virtually constant intensity distribution profile, with the intensity variation on the order of only 1%, with the intensity minimum around the middle of the film (Supplementary Fig. 1).

### Spin current simulations

Ferromagnetic 3*d* metals such as Fe are known to have a small, yet non-vanishing, inverse spin Hall effect (see e.g. ref. ^44^). To exclude that any spin current along the depth of the sample would lead to an electric THz emission via ISHE mechanism, we have performed rigorous simulations. Specifically, to make our simulations parameter-free, we only use ab initio computed quantities as input. We have therefore computed ab initio the SHE and ISHE coefficients of Fe and Pd; the computed spin Hall conductivities of bulk bcc Fe and fcc Pd are 14.96 and 700.93 (*ħ*/*e*) S cm^−1^, respectively, which implies that the computed spin Hall angle of Pd is 46.85 times larger than that of Fe (Salemi, L. & Oppeneer, P. M. unpublished). In addition we have used spin-dependent hot-electron lifetimes and velocities computed previously^45^. With these input quantities and using the ultrafast superdiffusion code^27^ we have simulated the charge and spin currents in MgO/Fe/MgO and MgO/Fe/Pd structures under the same excitation condition as in our experiment (800 nm, 100 fs pulse). The results (Supplementary Fig. 2) demonstrate that the electric dipole contribution from the MgO/Fe/MgO structure is extremely small and can be safely neglected, leaving the transient demagnetization as the only source of its THz emission.

### Magnetic and electric dipole radiation

The far-field electric field components of magnetic **E**_**M**_(*t*) and electric **E**_**P**_(*t*) dipole radiation from a point source^46^ read

1 $${{\mathbf{E}}_{\mathrm{M}}\left( t \right) = iFT\left\{ { - \frac{1}{{4\pi }}\sqrt {\frac{{\mu _0}}{{{\it{\epsilon }}_0}}} k^2\left( {{\mathbf{n}} \times {\mathbf{M}}} \right)\frac{{e^{ikr}}}{r}} \right\},}$$

2 $${{\mathbf{E}}_{\mathrm{P}}\left( t \right) = iFT\left\{ {\frac{1}{{4\pi {\it{\epsilon }}_0}}k^2\left( {{\mathbf{n}} \times {\mathbf{P}}} \right) \times {\mathbf{n}}\frac{{e^{ikr}}}{r}} \right\}.} $$

Here *iFT* stands for inverse Fourier transform, *μ*_0_ is the vacuum permeability, ${\it{\epsilon }}_0$ the vacuum permittivity, *k* is the wavevector, **n** the unit vector pointing towards the detecting position from the emitting dipole, **M** and **P** are, respectively, the frequency-dependent magnetization and polarization^20^ (i.e., Fourier transforms of *M*(*t*) and *P*(*t*)), and *r* is the distance from the emitting dipole to the position of detection.

### Symmetry distinction of THz emission

Two types of samples were prepared. Both of them were 10 nm ferromagnetic iron (Fe) films deposited on an insulating MgO substrate. One was capped with a 12 nm insulating MgO layer (MgO/Fe/MgO), and the other was capped with a 5 nm metallic Pd layer (MgO/Fe/Pd). The samples were fully magnetized in-plane along the *x*-axis (see Fig. 1) by an applied external magnetic field of 50 mT, and were excited by femtosecond laser pulses with a fluence of 1.1 mJ cm^−2^. First, both samples were pumped from the cap layer side, and the emitted THz waves were measured by FEOS in the far field, in the direction of the pump beam propagation. Second, we flipped the two samples while keeping the external magnetic field and the THz detection geometry unchanged, thus now pumping the samples from the substrate side. Finally, both samples were pumped at the cap layer, but the external magnetic field was reversed.

The experimental results (Supplementary Fig. 4) show that the polarity of THz emitted from MgO/Fe/MgO only depends on the external magnetic field, while both the external magnetic field and the geometry of the sample can affect the polarity of THz emitted from MgO/Fe/Pd. We conclude that the THz emission from MgO/Fe/MgO is due to the ultrafast magnetization dynamics, whereas the electric dipole radiation caused by the spin-charge conversion dominates the THz emission in MgO/Fe/Pd.

### Reconstruction of magnetization dynamics

We performed direct experimental characterization, fully supported by the theoretical modeling, of the THz field propagation function in our experiments, rigorously relating the THz EOS measured in the far-field, to its source, the magnetization dynamics in the sample *M*(*t*). For this we used a reference electric-dipole THz emitter, a 0.5-mm-thick ZnTe crystal, in place of MgO/Fe/MgO sample. As a result, we achieve precise reconstruction of the fully calibrated transfer function of our experiment, relating the dynamics of polarization *P*(*t*) or magnetization *M*(*t*) at the surface of the laser-excited THz emitter to our observable, the detected THz EOS.

The basis for the reconstruction protocol is the accurate establishment of the complete transfer function of the experiment in the frequency domain, which includes (i) conversion of time-dependent magnetization (polarization) in the sample into electromagnetic radiation *f*_rad,M_(*ω*) and *f*_rad,P_(*ω*), (ii) propagation of this electromagnetic radiation in the spectrometer towards detector crystal *f*_prop_(*ω*), and (iii) detection by FEOS of the electromagnetic field in the detector crystal *f*_d_(*ω*).

While the radiative functions *f*_rad,M_(*ω*) and *f*_rad,P_(*ω*) are different for magnetic dipole (MgO/Fe/MgO) and electric dipole (ZnTe) emitters, the propagation *f*_prop_(*ω*) and detection *f*_d_(*ω*) functions remain identical for both cases.

The total detected EOS for magnetic and electric dipole emission cases, *S*_M_(*ω*) and *S*_P_(*ω*) respectively, are

3 $${S_{\mathrm{M}}\left( \omega \right) = M\left( \omega \right)f_{{\mathrm{rad}},{\mathrm{M}}}\left( \omega \right)f_{{\mathrm{prop}}}\left( \omega \right)f_{\mathrm{d}}\left( \omega \right),} $$

4 $${S_{\mathrm{P}}\left( \omega \right) = P\left( \omega \right)f_{{\mathrm{rad}},{\mathrm{P}}}\left( \omega \right)f_{{\mathrm{prop}}}\left( \omega \right)f_{\mathrm{d}}\left( \omega \right),} $$

where *M*(*ω*) and *P*(*ω*) are the frequency-dependent magnetization and polarization, respectively (i.e., the Fourier trasnsforms of *M*(*t*) and *P*(*t*)).

The reconstruction of the quantity of interest *M*(*ω*) from the observable, EOS S_M_(*ω*) is therefore

5 $$M\left( \omega \right) = \frac{{S_{\mathrm{M}}\left( \omega \right)}}{{f_{{\mathrm{rad}},{\mathrm{M}}}\left( \omega \right)f_{{\mathrm{prop}}}\left( \omega \right)f_{\mathrm{d}}\left( \omega \right)}}.$$

The radiative functions *f*_rad,M_(*ω*) = *FT*(*E*_M_(*t*)) and *f*_rad,P_(*ω*) = *FT*(*E*_P_(*t*)) are given by Eqs. (1) and (2).

In order to experimentally establish the spectrometer function, i.e., the propagation and detection function of our experiment *f*_prop_(*ω*)*f*_d_(*ω*), we use a reference electric dipole emitter, a 0.5-mm-thick <110> ZnTe crystal. The process of THz generation by optical rectification of femtosecond laser pulses in transparent nonlinear crystals is by now very well understood, and can be modeled quantitatively very accurately from the first principles, using as an input the temporal intensity profile of the laser pulse, the value for electro-optic coefficient of the crystal, and the THz dispersion of its complex-valued refractive index (see e.g. ref. ^47^ for the comprehensive description of the calculation). This allows one to very accurately calculate the polarization at the outcoupling crystal surface, i.e., the parameter *P*(*ω*) in Eq. (4). With the knowledge of *P*(*ω*), it is therefore straightforward to recover the complete propagation and detection function of our experiment *f*_prop_(*ω*)*f*_d_(*ω*), from the measured EOS of the THz emission of a reference electric dipole emitter *S*_P_(*ω*)^48^.

Now that the spectrometer function *f*_prop_(*ω*)*f*_d_(*ω*) is established, it can be directly applied to the reconstruction of the magnetization dynamics *M*(*t*) in our magnetic dipole emitter MgO/Fe/MgO, from the correspondingly measured EOS *S*_M_(*t*), using the inverse Fourier transform of the Eq. (5). This yields the recovered, and fully calibrated, magnetization dynamics *M*(*t*) in our experiment, shown in Figs. 2 and 3 of the main text.

### Incoherent excitation of magnons

Although experimentally we cannot uniquely resolve which microscopic mechanism leads to the observed magnetization dynamics, in this section we show that incoherent excitation of magnons is the only plausible mechanism responsible for the ultrafast magnetization dynamics in the MgO/Fe/MgO structure.

On the one hand, it has been recently demonstrated^11,32,33^ that the incoherent excitation of magnon modes is a dominant mechanism contributing to ultrafast demagnetization. Moreover, Mlynczak et al.^49^ have recently shown that the electron-magnon scattering process is a very relevant scattering mechanism in Fe.

On the other hand, transport mechanisms, such as spin superdiffusion or heat transport, can be disregarded as shown by the simulations. Coherent processes stemming from the direct light-spin interaction can also be neglected due to the linear light polarization. Another relevant mechanism which could potentially explain the transient magnetization dynamics is the Elliott-Yafet mechanism, in which the electrons transfer angular momentum to the phonons. However, it has been theoretically shown that it only provides a small contribution to the ultrafast demagnetization in ferromagnetic 3*d* metals^40^.

Another plausible mechanism that has gained attention in the last years, is the spin-flip Coulomb scattering model, which is based on the ultrafast transfer of angular momentum from the electronic spin to the electronic orbital angular momentum, and subsequently to the lattice^50^. However, the lack of a theoretical model to explain the ultrafast (tens of femtoseconds) transfer of angular momentum from the electronic orbitals to the lattice, and the relatively slow demagnetization time found in our experiments (around 500 fs) suggest that this theoretically proposal is also not relevant in our experiment. As we show below, excitation of incoherent magnons by electron-magnon scattering can perfectly well explain our experimental observations.

### Microscopic multi-temperature model

To simulate the transient magnetization dynamics *M*(*t*) in Fe we use a MMTM, which describes the magnetization dynamics as an incoherent excitation of magnon modes through a complex interplay between electrons, spin waves and the lattice. Our model goes well beyond the usual three-temperature model (3TM) and provides an out-of-equilibrium description of electron and lattice dynamics with an explicit inclusion of phonon-phonon scattering through an anharmonic phonon interaction term^51^. Mathematically, the temporal evolution of the three different subsystems is modeled by the following set of coupled differential equations

6 $$C_{\mathrm{e}}\frac{{\partial T_{\mathrm{e}}}}{{\partial t}} 	= G\left( {T_{\mathrm{s}} - T_{\mathrm{e}}} \right) + \mathop {\sum }\limits_Q C_Q\gamma _QI\left( {T_{\mathrm{e}}} \right)\left( {T_{\mathrm{l}}^Q - T_{\mathrm{e}}} \right)\left[ {1 + J\left( {\omega _Q,T_{\mathrm{l}}^Q} \right)\left( {T_{\mathrm{l}}^Q - T_{\mathrm{e}}} \right)} \right]\\ 	\quad+ \frac{{\partial U_{{\mathrm{e}} - {\mathrm{e}}}}}{{\partial t}},$$

7 $$C_Q\frac{{\partial T_{\mathrm{l}}^Q}}{{\partial t}} 	= - C_Q\gamma _QI\left( {T_{\mathrm{e}}} \right)\left( {T_{\mathrm{l}}^Q - T_{\mathrm{e}}} \right)\left[ {1 + J\left( {\omega _Q,T_{\mathrm{l}}^Q} \right)\left( {T_{\mathrm{l}}^Q - T_{\mathrm{e}}} \right)} \right]\\ 	\quad - \mathop {\sum}\limits_k {C_Q} \Gamma _{Qk}\left( {T_{\mathrm{l}}^Q - T_{\mathrm{l}}^k} \right)\left[ {1 + J\left( {\omega _Q,T_{\mathrm{l}}^Q} \right)\left( {T_{\mathrm{l}}^Q - T_{\mathrm{l}}^k} \right)} \right]\\ 	\quad+ \frac{{\partial U_{{\mathrm{e}} - {\mathrm{ph}}}}}{{\partial t}},\,\,for\,Q = Q_1, \cdots ,Q_N,$$

8 $$ C_{\mathrm{s}}\frac{{\partial T_{\mathrm{s}}}}{{\partial t}} = - G\left( {T_{\mathrm{s}} - T_{\mathrm{e}}} \right).\qquad$$

Here *T*_e_, *T*_s_, and $T_{\mathrm{l}}^Q$ are the electron, spin and phonon mode-dependent temperatures. *Q* accounts for the *N* distinct and independent phonon subsystems, each of them corresponding to a specific branch, *υ*, and momentum, *q*, having different temperatures $T_{\mathrm{l}}^Q$. *C*_e_, *C*_s_, and *C*_*Q*_ are the temperature-dependent electronic, spin and phonon mode-dependent heat capacities, respectively*. γ*_*Q*_ and Γ_*Qk*_ are the mode-dependent linewidth due to electron-phonon and phonon-phonon interactions, respectively. *G* is the electron-magnon coupling constant. *I*(*T*_e_) accounts for the dependence of the electron-phonon coupling on the electronic temperature. $\frac{{\partial U_{{\mathrm{e}} - {\mathrm{ph}}}}}{{\partial t}}$ and $\frac{{\partial U_{{\mathrm{e}} - {\mathrm{e}}}}}{{\partial t}}$ describe the energy transfer rate from the laser-induced nonequlibrium electrons to the lattice through electron-phonon interaction and into thermal electrons via electron-electron interactions, respectively. $J\left( {\omega _Q,T_{\mathrm{l}}^Q} \right)$ is the second-order Taylor expansion of the out-of-equilibrium phonon populations around the mode-dependent phonon temperatures (see ref. ^51^ for full forms). The effective spin temperature, *T*_s_, is in our model equivalent to the magnon temperature, which characterizes the incoherent excitation of magnon modes in the 3TM.

It is worth mentioning that, while the model for the out-of-equilibrium description of the electron and lattice dynamics has been derived from a microscopic theory and is therefore fit-free (the parameters are ab initio computed), the coupling of these two subsystems with the spin system is introduced phenomenologically, and the electron-magnon coupling constant is chosen to reproduce the experimental demagnetization time. In addition, a correct treatment of the spin system would need to include effective mode- and *q*-dependent spin temperatures to account for the distinct *q*-dependent electron-magnon scatterings. However, currently there does not exist a reliable theoretical model to provide such *q*-dependent electron-magnon coupling, and therefore such description is beyond our present capabilities. Notwithstanding, we can effectively treat the global effect of heating the magnon system by considering the spin system to be individually thermalized and work with an effective electron-magnon coupling, which is then chosen to reproduce the experimental demagnetization time.

From the MMTM model we determine the time evolution of the spin temperature which then is used in the analytic form of the temperature-dependent magnetization for bcc Fe

9 $$ {M\left( {T_{\mathrm{s}}} \right) = M\left( 0 \right)\left( {1 - \frac{{T_{\mathrm{s}}}}{{T_{\mathrm{c}}}}} \right)^{0.39},} $$

where *T*_c_ = 1044 K is the Curie temperature of Fe.

In addition, the transient phonon-mode dependent lattice temperatures are used to model the strain dynamics, as outlined in the following.

### Lattice strain dynamics

We consider three different mechanisms that contribute to the creation of strain in the lattice. These mechanisms are: first, the force induced by the optically excited electrons; second, the force induced by the ultrafast demagnetization (a new electronic surface potential is generated following the magnon emission); and third, the thermal strain due to heating of the lattice. The first and second processes originate from the deformation potential mechanism^52^ and happen at a time scale below 100 fs, where the strain caused by acoustic phonons depends on the deformation potential and the absorbed laser energy. The third process is induced by the changing temperature distribution in a thermoelastic model wherein a linear relation is established between the stress pulse and the varying temperature distribution. Although in the latter process the changing electronic and lattice temperatures contribute to the stress pulse, the regime of small laser intensities used in the experiment along with the smallness of the electron heat capacity as compared with the lattice one, allows one to neglect the electronic contribution.

The transient lattice strain along the direction perpendicular to the film (*z*) can then be written as

10 $$\eta \left( {z,t} \right) = - \frac{1}{{2\rho v^{2}}}\sum_{Q} \int_{ - \infty }^{ + \infty } g_{Q} C_{Q} {\mathrm{sgn}}\left(z - v\left(t - t^{\prime} \right) \right)\left. {\frac{{\partial T_{\mathrm{l}}^Q}}{{\partial t}}} \right|_{\left( {\left| {z - v\left( {t - t^{\prime} } \right)} \right|,t^{\prime} } \right)}{{\mathrm{d}}t}^{\prime},$$

where *ρ* is the density and *v* the sound velocity of Fe, and *g*_*Q*_ is the mode-dependent Grüneisen parameter, which are computed from first principles. The term $\frac{{\partial T_{\mathrm{l}}^Q}}{{\partial t}}$ is determined by the solution of the MMTM (see above). Thus, Eq. (10) is a modified out-of-equilibrium version of the expression provided by Wright^53^. Although it may provide the same total expansion of the lattice, it is reached through different strain dynamics. Notably, the solution of the MMTM yields that the time evolution of the strain produced by heating covers a range of several picoseconds.

The different time scales of the three different processes that induce the strain pulses enable a simplification in the modeling. Thus, strain pulses generated by the deformation potential mechanisms are assumed to happen instantaneously following laser excitation with an amplitude proportional to the laser excitation, which adds to the heating induced strain.

Schematically, the process is as follows: At each of the two Fe/MgO interfaces a tensile strain is generated following laser excitation. These pulses travel within the Fe layer toward the other interface at the Fe sound velocity (5060.05 m s^−1^). Correspondingly, a compressive strain is generated at the Fe/MgO interface which travels within the MgO system at the MgO sound velocity (9100 m s^−1^). When the strains traveling within the Fe film reach one of the Fe/MgO interfaces, the pulses become partially reflected (92.5% transmission, due to the different impedances between Fe and MgO) inducing a change of polarization of the strain (tensile waves become compressive waves and vice versa), while the rest goes into the MgO films. On the other hand, the strain waves are completely reflected on the MgO/air interfaces. At the end of the simulation, a finite strain remains in the system due to the heating effect that induces the thermal expansion of the system.

To recover the normal volume expansion (in our case normal surface expansion, *u*(*t*)) we integrate over the whole Fe film thickness the time-dependent strain pulses, getting

11 $$u\left( t \right) = \int_0^{z_{{\mathrm{max}}}} \eta \left( {z,t} \right){{\mathrm{d}}z},$$

where *z*_max_ = 10 nm. The calculated time evolution of the system’s expansion is shown in Fig. 2d for a laser excitation of 1.02 mJ cm^−2^.

### Magnetization dynamics

The final magnetization dynamics is calculated by combining the MMTM and the strain dynamics. Thus, we can write

12 $$ {\frac{{M\left( {T_{\mathrm{s}}} \right)}}{{M\left( {T_0} \right)}} = \left( {\frac{{T_{\mathrm{c}} - T_{\mathrm{s}}}}{{T_{\mathrm{c}} - T_0}}} \right)^{0.39} + \Delta M\left( {u\left( t \right)} \right) + f_{{\mathrm{recovery}}},} $$

where *f*_recovery_ is a phenomenological function linear in time, introduced to recover the long-time behavior of the magnetization dynamics (relaxation back to equilibrium), and which accounts for the physical processes that are not included in our model, such as phonon-magnon coupling or heat diffusion, which are relevant at long-time scales. Δ*M*(*u*(*t*)) is a function that represents the change of magnetization due to the change of the system’s volume. It is driven by the modulation of exchange interaction in iron, and phenomenologically, can be written as:

13 $$ {\Delta M\left( {u\left( t \right)} \right) = \Delta J\left( {u\left( t \right)} \right)\frac{1}{2}\frac{{M_0}}{{J_0}},} $$

where the factor 1/2 comes from the fact that around equilibrium position a 2% change of *J* produces a 1% change of magnetization^54,55^

We also assume that

14 $$ {\Delta J\left( {u\left( t \right)} \right) = \frac{{\mathrm{d}}J}{{\mathrm{d}}u}u\left( t \right),} $$

where *u*(*t*) is the system elongation in *z*-direction (see Eq. (11)). By combining Eqs. (13) and (14) we obtain

15 $$ {\Delta M\left( {u\left( t \right)} \right) = \frac{{\mathrm{d}}J}{{\mathrm{d}}u}u\left( t \right)\frac{1}{2}\frac{{M_0}}{{J_0}},} $$

where the value of the gradient of the exchange constant is fitted to provide the experimentally observed transient magnetization dynamics. For the specific case of Fe we obtain

16 $${\Delta M\left( {u\left( t \right)} \right) = 80.88\frac{{\mu _{\mathrm{B}}}}{{\mathrm{{\AA}}}}u\left( t \right).} $$

This expression is used along with the elongation values provided by Eq. (11) to obtain the transient magnetization driven by the acoustic waves in Eq. (12). As example, a system’s elongation of 40 fm would induce an increase of magnetization of the order of 0.16% of the equilibrium magnetization, as can be seen in Fig. 2b, d for a delay time between 3 and 4.5 ps. On the other hand, lattice strains of the order of 10^−3^ would induce magnetization changes of the order of 37% of the equilibrium magnetization. However, it is important to mention that although those large lattice strains are experimentally accessible at short time scales, their effect on the transient magnetization might be largely shadowed by the more dominant magnetization dynamics induced by hot magnons. In addition, large elongations would introduce nonlinear effects in the above relation, and also significant changes in the magnetic anisotropy, leading to a distinct magnetization dynamics.

The value of equilibrium magnetization in iron *μ*_0_*M*_0_ = 2.15 T^56–58^ was used in the calculations of relative magnetization dynamics.

### Sample preparation

The iron films were deposited on double polished MgO <100> substrates and capped with 12 nm of MgO and 5 nm of Pd at room temperature, respectively. The molecular beam epitaxy was performed to deposit 10 nm of Fe at room temperature with subsequent 90 minutes heat treatment at 800 K, necessary to obtain highly ordered monocrystalline Fe layer. The deposition rate of Fe layer was 0.05 nm min^−1^ at standard 10^−10^ Torr (10^−8^ Pa) pressure under reflection high energy electron diffraction control to verify the crystal quality. The thicknesses were controlled in situ by quartz balance sensing.

## Supplementary information

Supplementary Information

## Data Availability

The data that support the findings of this study are available from the corresponding author upon reasonable request.

## References

[1] Stöhr, J. & Siegmann, H. C. *Magnetism From Fundamentals to Nanoscale Dynamics*. (Springer-Verlag, Berlin Heidelberg, 2006).

[2] Chappert C, Fert A, Van Dau FN (2007). The emergence of spin electronics in data storage. Nat. Mater..

[3] Parkin SSP, Hayashi M, Thomas L (2008). Magnetic domain-wall racetrack memory. Science.

[4] Chumak AV, Vasyuchka VI, Serga AA, Hillebrands B (2015). Magnon spintronics. Nat. Phys..

[5] Cornelissen LJ, Liu J, Duine RA, Ben Youssef J, Van Wees BJ (2015). Long-distance transport of magnon spin information in a magnetic insulator at room temperature. Nat. Phys..

[6] Beaurepaire E, Merle J-C, Daunois A, Bigot J-Y (1996). Ultrafast spin dynamics in ferromagnetic nickel. Phys. Rev. Lett..

[7] Dornes C (2019). The ultrafast Einstein–de Haas effect. Nature.

[8] Walowski J, Münzenberg M (2016). Perspective: ultrafast magnetism and THz spintronics. J. Appl. Phys..

[9] Eschenlohr A, Bovensiepen U (2018). Special issue on ultrafast magnetism. J. Phys. Condens. Matter.

[10] Cinchetti M (2009). Determination of spin injection and transport in a ferromagnet/organic semiconductor heterojunction by two-photon photoemission. Nat. Mater..

[11] Eich S (2017). Band structure evolution during the ultrafast ferromagnetic-paramagnetic phase transition in cobalt. Sci. Adv..

[12] Wieczorek J (2015). Separation of ultrafast spin currents and spin-flip scattering in Co/Cu(001) driven by femtosecond laser excitation employing the complex magneto-optical Kerr effect. Phys. Rev. B.

[13] Razdolski I (2017). Analysis of the time-resolved magneto-optical Kerr effect for ultrafast magnetization dynamics in ferromagnetic thin films. J. Phys. Condens. Matter.

[14] Melnikov A, Razdolski I, Wehling T (2011). Ultrafast transport of laser-excited spin-polarized carriers in Au/Fe/MgO (001). Phys. Rev. Lett..

[15] Beaurepaire E (2004). Coherent terahertz emission from ferromagnetic films excited by femtosecond laser pulses. Appl. Phys. Lett..

[16] Hilton DJ (2004). Terahertz emission via ultrashort-pulse excitation of magnetic metal films. Opt. Lett..

[17] Kampfrath T (2013). Terahertz spin current pulses controlled by magnetic heterostructures. Nat. Nanotechnol..

[18] Huisman TJ, Mikhaylovskiy RV, Tsukamoto A, Rasing T, Kimel AV (2015). Simultaneous measurements of terahertz emission and magneto-optical Kerr effect for resolving ultrafast laser-induced demagnetization dynamics. Phys. Rev. B.

[19] Seifert T (2016). Efficient metallic spintronic emitters of ultrabroadband terahertz radiation. Nat. Photonics.

[20] Huisman TJ (2016). Femtosecond control of electric currents in metallic ferromagnetic heterostructures. Nat. Nanotechnol..

[21] Wu Y (2017). High-performance THz emitters based on ferromagnetic/nonmagnetic heterostructures. Adv. Mater..

[22] Yamaguchi K, Nakajima M, Suemoto T (2010). Coherent control of spin precession motion with impulsive magnetic fields of half-cycle terahertz radiation. Phys. Rev. Lett..

[23] Jin Z (2013). Single-pulse terahertz coherent control of spin resonance in the canted antiferromagnet YFeO_3_, mediated by dielectric anisotropy. Phys. Rev. B.

[24] Li X (2018). Observation of Dicke cooperativity in magnetic interactions. Science.

[25] Jin Z (2015). Accessing the fundamentals of magnetotransport in metals with terahertz probes. Nat. Phys..

[26] Gallot G, Grischkowsky D (1999). Electro-optic detection of terahertz radiation. J. Opt. Soc. Am. B.

[27] Battiato M, Carva K, Oppeneer PM (2010). Superdiffusive spin transport as a mechanism of ultrafast demagnetization. Phys. Rev. Lett..

[28] Mics Z, D’Angio A, Jensen SA, Bonn M, Turchinovich D (2013). Density-dependent electron scattering in photoexcited GaAs in strongly diffusive regime. Appl. Phys. Lett..

[29] Mics Z (2015). Thermodynamic picture of ultrafast charge transport in graphene. Nat. Commun..

[30] Turchinovich D, D’Angelo F, Bonn M (2017). Femtosecond-timescale buildup of electron mobility in GaAs observed via ultrabroadband transient terahertz spectroscopy. Appl. Phys. Lett..

[31] Seifert TS (2018). Terahertz spectroscopy for all-optical spintronic characterization of the spin-Hall-effect metals Pt, W and Cu 80 Ir 20. J. Phys. D. Appl. Phys..

[32] Schmidt AB (2010). Ultrafast magnon generation in an Fe film on Cu(100). Phys. Rev. Lett..

[33] Turgut E (2016). Stoner versus Heisenberg: Ultrafast exchange reduction and magnon generation during laser-induced demagnetization. Phys. Rev. B.

[34] Henighan T (2016). Generation mechanism of terahertz coherent acoustic phonons in Fe. Phys. Rev. B.

[35] van Capel PJS (2011). Correlated terahertz acoustic and electromagnetic emission in dynamically screened InGaN/GaN quantum wells. Phys. Rev. B.

[36] Kim JW, Vomir M, Bigot JY (2012). Ultrafast magnetoacoustics in nickel films. Phys. Rev. Lett..

[37] Willig L (2019). Finite-size effects in ultrafast remagnetization dynamics of FePt. Phys. Rev. B.

[38] Maehrlein SF (2018). Dissecting spin-phonon equilibration in ferrimagnetic insulators by ultrafast lattice excitation. Sci. Adv..

[39] Koopmans B (2010). Explaining the paradoxical diversity of ultrafast laser-induced demagnetization. Nat. Mater..

[40] Carva K, Battiato M, Oppeneer PM (2011). Ab initio investigation of the Elliott-Yafet electron-phonon mechanism in laser-induced ultrafast demagnetization. Phys. Rev. Lett..

[41] Maldonado P, Kvashnin YO (2019). Microscopic theory of ultrafast out-of-equilibrium magnon-phonon dynamics in insulators. Phys. Rev. B.

[42] Shinde, S. L. & Srivastava, G. P. eds. *Length-Scale Dependent Phonon Interactions*. (Springer, New York, 2014)..

[43] Johnson PB, Christy RW (1974). Optical constants of transition metals: Ti, V, Cr, Mn, Fe, Co, Ni, and Pd. Phys. Rev. B.

[44] Miao BF, Huang SY, Qu D, Chien CL (2013). Inverse spin Hall effect in a ferromagnetic metal. Phys. Rev. Lett..

[45] Zhukov VP, Chulkov EV, Echenique PM (2006). Lifetimes and inelastic mean free path of low-energy excited electrons in Fe, Ni, Pt, and Au: Ab initio GW+T calculations. Phys. Rev. B.

[46] Jackson, J. D. *Classical Electrodynamics* (John Wiley & Sons, Inc., 1999).

[47] Faure J, Van Tilborg J, Kaindl RA, Leemans WP (2004). Modelling laser-based table-top THz sources: optical rectification, propagation and electro-optic sampling. Opt. Quantum Electron..

[48] Seifert TS (2018). Femtosecond formation dynamics of the spin Seebeck effect revealed by terahertz spectroscopy. Nat. Commun..

[49] Młyńczak E (2019). Kink far below the Fermi level reveals new electron-magnon scattering channel in Fe. Nat. Commun..

[50] Töws W, Pastor GM (2015). Many-body theory of ultrafast demagnetization and angular momentum transfer in ferromagnetic transition metals. Phys. Rev. Lett..

[51] Maldonado P, Carva K, Flammer M, Oppeneer PM (2017). Theory of out-of-equilibrium ultrafast relaxation dynamics in metals. Phys. Rev. B.

[52] Yu, P. Y. & Cardona, M. *Fundamentals of Semiconductors: Physics and Material Properties* (Springer, 2005).

[53] Wright OB (1994). Ultrafast nonequilibrium stress generation in gold and silver. Phys. Rev. B.

[54] Wang H, Ma PW, Woo CH (2010). Exchange interaction function for spin-lattice coupling in bcc iron. Phys. Rev. B.

[55] Kvashnin YO (2016). Microscopic origin of Heisenberg and non-Heisenberg exchange interactions in ferromagnetic bcc Fe. Phys. Rev. Lett..

[56] Building H (1971). The magnetization of pure iron and nickel. Proc. R. Soc. Lond. A..

[57] Sun L, Hao Y, Chien CL, Searson PC, Searson PC (2005). Tuning the properties of magnetic nanowires. IBM J. Res. Dev..

[58] Weber M, Koch R, Rieder KH (1994). UHV cantilever beam technique for quantitative measurements of magnetization, magnetostriction, and intrinsic stress of ultrathin magnetic films. Phys. Rev. Lett..

