# Supplementary material for: Ultrafast terahertz magnetometry
Source: Nat Commun. 2020 Aug 25;11:4247. doi: 10.1038/s41467-020-17935-6 (PMC7447779; doi:10.1038/s41467-020-17935-6)
Supplement: Supplementary file 1 — Supplementary Information [file 41467_2020_17935_MOESM1_ESM.pdf]

Supplementary Information

Ultrafast Terahertz Magnetometry

Zhang et al.

Supplementary Note 1: Pump intensity distribution within the iron film

In Fig. 1 we show the calculated optical intensity distribution within our MgO-capped 10 nm film, performed using two different methods, the transfer matrix formalism, and purely numerically using COMSOL(c), showing excellent agreement with one another.

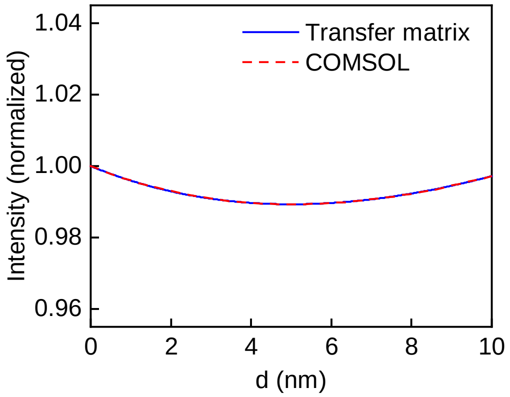

Supplementary Figure 1. Intensity distribution of 800 nm pump light within a 10 nm thick capped Fe film. The intensity distribution was calculated using the transfer matrix formalism (blue solid line) and was also numerically simulated using COMSOL(c) (red dashed line).

Supplementary Note 2: Spin current simulations

The simulation results of charge and spin currents in MgO/Fe/MgO and MgO/Fe/Pd structures under the same excitation condition as in our experiment (800 nm, 100 fs pulse), shown in Fig. 2, demonstrate that the electric dipole contribution from the MgO/Fe/MgO structure is extremely small and can be safely neglected, leaving the transient demagnetization as the only source of its THz emission.

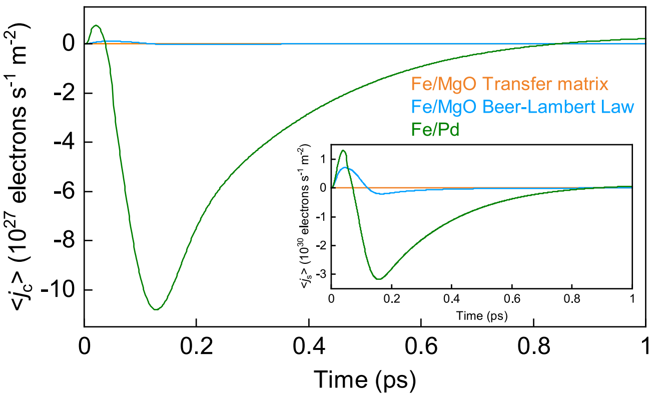

Supplementary Figure 2. Comparison of simulated charge j_c and spin j_s currents in MgO/Fe/Pd (green line) and MgO/Fe/MgO (blue and orange lines). The blue line shows the worst case scenario of bulk-like pump absorption in the Fe film according to the Beer-Lambert law. The orange line is calculated for the realistic scenario of quasi-uniform pump absorption in Fe film, as shown in Fig. 1. These calculations demonstrate that ISHE in MgO/Fe/MgO structure is negligible, leaving the transient demagnetization due to local quench of magnetic order under the laser excitation as the only possible source of the THz emission from this structure.

Supplementary Note 3: Further details of symmetry distinction of THz emission

Two types of samples were prepared. Both of them were 10 nm ferromagnetic iron (Fe) films deposited on an insulating MgO substrate. One was capped with a 12 nm insulating MgO layer (MgO/Fe/MgO, Fig. 3 ①③⑤), and the other was capped with a 5 nm metallic Pd layer (MgO/Fe/Pd, Fig. 3 ②④⑥). For the MgO/Fe/MgO sample, the femtosecond laser excitation induces an ultrafast quenching of magnetic order in the Fe film, leading to the THz emission, which is corresponding to a magnetic dipole radiation $E_{\mathrm{THz}} \propto \partial^2 M/\partial t^2$ [1]. For the MgO/Fe/Pd sample, after the laser excitation, the superdiffusive spin-polarized electrons from Fe layer propagate into the Pd layer [2], forming a short spin current pulse, which is partly deflected in the Pd layer due to the ISHE, and converted into an in-plane charge current, leading to the THz emission [3]. The relations between the THz electric field, the spin current, and the charge current is given by $\mathbf{j}_{\mathrm{c}} \propto \mathbf{j}_{\mathrm{s}} \times \mathbf{M}$ and $E_{\mathrm{THz}} \propto \partial j_c/\partial t$, corresponding to an electric dipole radiation. The magnetization $\mathbf{M}$ and the spin current $\mathbf{j}_{\mathrm{s}}$ can be experimentally controlled by changing the external magnetic field and the geometry of the sample.

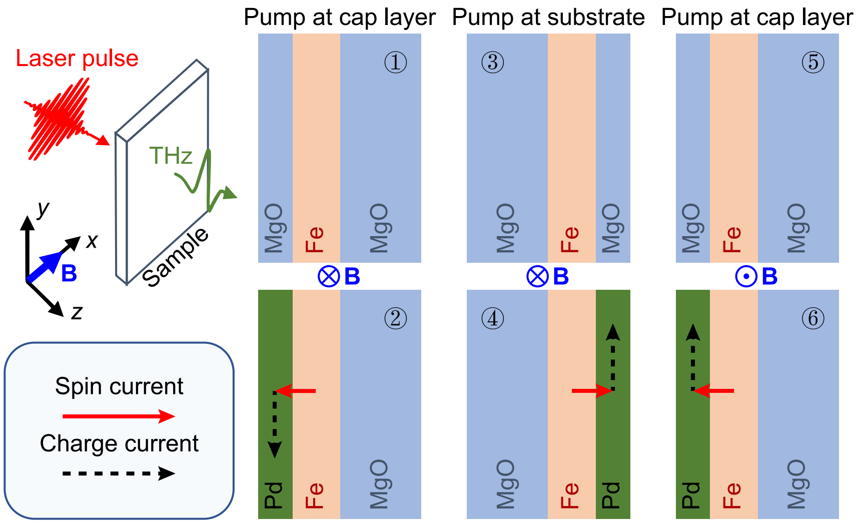

Supplementary Figure 3. Schematic for the terahertz emission and the laser excitation geometry. The MgO/Fe/MgO and MgO/Fe/Pd samples are fully magnetized in-plane along the *x*-axis by an external magnetic field, and are excited by 800 nm, 100 fs laser pulses, leading to the THz emission. In MgO/Fe/Pd sample, a spin current (red solid arrows) is induced and converted into a change current via ISHE (black dashed arrows). In ①② the samples are pumped from the cap layer side, in ③④ the samples are pumped from the substrate side, and in ⑤⑥ the samples are pumped from the cap layer side with the magnetic field applied in opposite direction as compared to ①②.

In the experiment, the samples were fully magnetized by an applied external magnetic field of 50 mT, which was parallel to the *x*-axis (in-plane), and were excited by femtosecond laser pulses with a fluence of 1.1 mJ cm^{-2}. First, both samples were pumped from the cap layer side (Fig. 3 ①②), and the emitted THz waves were measured by free-space electro-optic sampling (FEOS) in the far field, in the direction of the pump beam propagation. The blue and red solid lines in Fig. 4a,b show the measured electro-optic (EO) signals, respectively. Second, we flipped the

two samples while keeping the external magnetic field and the THz detection geometry unchanged (Fig. 3 ③④), thus now pumping the samples from the substrate side. The detected THz waves from this measurement are shown as blue and red dashed lines in Fig. 4a (note that blue solid ① and dashed ③ lines coincide in Fig. 4a). Finally, both samples were pumped at the cap layer, but the external magnetic field was reversed (Fig. 3 ⑤⑥), the measured THz emissions from two samples are shown as the blue and red dashed lines in Fig. 4b.

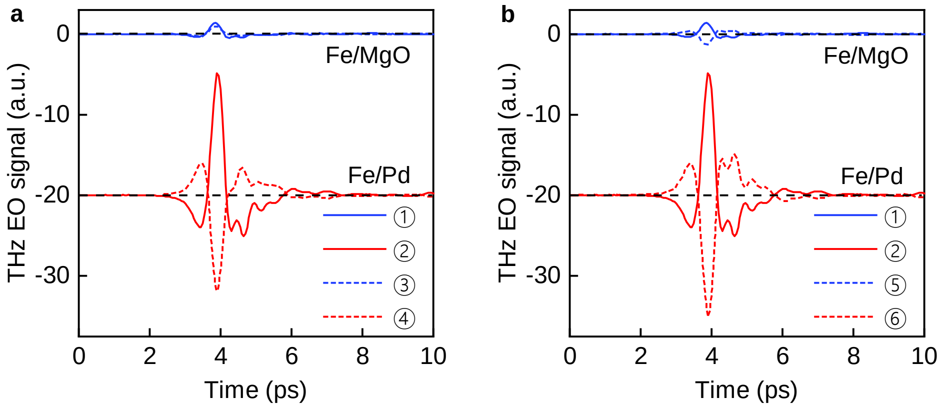

Supplementary Figure 4. THz EO signals from the MgO/Fe/MgO and MgO/Fe/Pd samples in different excitation geometries. **a**, MgO/Fe/MgO and MgO/Fe/Pd samples are pumped from the cap layer side (blue and red solid lines), and from the substrate side (blue and red dashed lines). **b**, MgO/Fe/MgO and MgO/Fe/Pd samples are pumped from the cap layer side with magnetic field along +*x* direction (blue and red solid lines) and along -*x* direction (blue and red dashed lines). ①②③④⑤⑥ correspond to the laser excitation geometries as shown in Fig. 3.

In Fig. 5 we show the noise level in our experiment in the time and frequency domains, as well as the acceptance bandwidth of our spectrometer (tested using the reference ZnTe emitter), clearly exceeding the bandwidth of the THz emission from MgO/Fe/MgO and MgO/Fe/Pd samples. Further, we demonstrate that the emission from MgO/Fe/MgO and MgO/Fe/Pd samples is free from atmospheric water absorption lines, which could otherwise compromise the signal reconstruction.

From the experimental results, we can see that the polarity of THz emitted from MgO/Fe/MgO only depends on the external magnetic field, while both the external magnetic field and the geometry of the sample can affect the polarity of THz emitted from MgO/Fe/Pd. We conclude that the THz emission from MgO/Fe/MgO is due to the ultrafast magnetization dynamics, whereas the electric dipole radiation caused by the spin-charge conversion dominates the THz emission in MgO/Fe/Pd. It should be noted that the spectrometer used here had an intermediate focus, therefore the shape of THz EO signals is different from our main experiment. Yet, this does not affect neither relative amplitude nor the phase of the THz EO signals discussed in this section, and hence does not affect the validity of our conclusions.

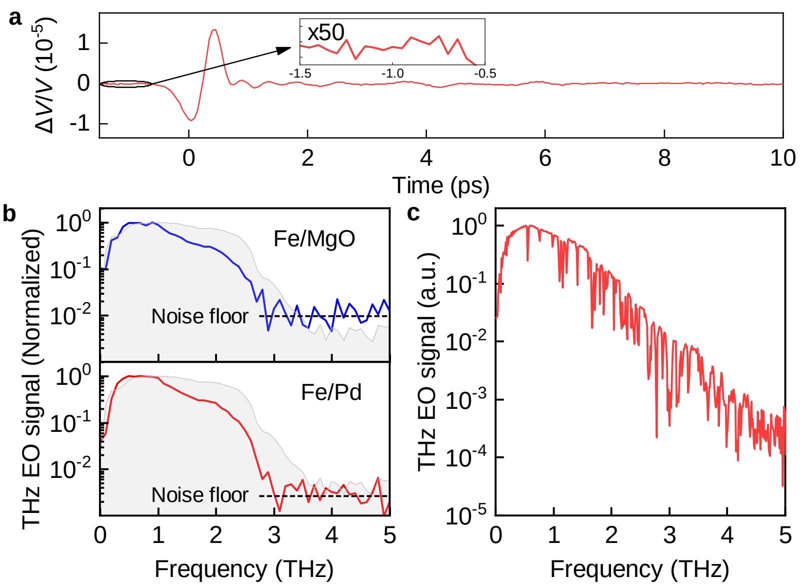

Supplementary Figure 5. The noise level in THz FEOS signal, the emitted THz bandwidth, the acceptance bandwidth of the THz detector, and the positions of water lines in the THz spectrum. **a,** Measured FEOS signal of the THz emission from MgO/Fe/MgO sample, under the excitation of 0.51 mJ cm^{-2}. Inset, zoomed FEOS signal in the range from -1.5 ps to -0.5 ps, showing the true noise fluctuations in the measurement. **b,** The measured THz spectra of MgO/Fe/MgO and MgO/Fe/Pd emission. Grey lines indicate the spectra of the THz emission from 0.5 mm ZnTe reference emitter, detected in 1 mm ZnTe detector crystal, used universally for THz detection in this work. The acceptance bandwidth of a 1 mm ZnTe crystal clearly exceeds that of both MgO/Fe/MgO and MgO/Fe/Pd samples, thus making the signal reconstruction possible. Noise floor is indicated. **c**, THz spectrum measured in lab air (i.e. without dry nitrogen purging), showing the typical water absorption lines. Note the absence of the water lines in measured MgO/Fe/MgO and MgO/Fe/Pd spectra **b**, demonstrating that the nitrogen purging level in our experiment is sufficient, and the possible presence of water lines could not compromise our analysis.

Supplementary Note 4: Further details of reconstruction of magnetization dynamics

In order to ensure that we indeed do understand our experiment to a sufficient detail, additionally we performed a full simulation of the complete process of THz generation, propagation, and electro-optic sampling of the electric dipole radiation of our reference THz emitter, the 0.5 mm – thick <110> ZnTe crystal. Apart from the generation and electro-optic sampling [4], the effects of THz frequency filtering in the optical pump beam block, as well as the frequency-dependent diffraction of the THz beam in the spectrometer were included. The results of the experiment and calculation are presented in Fig. 6a, demonstrating a very good agreement with one another. In order to quantify the accuracy of our modeling, we have established the frequency-dependent non-ideality factor $S_{\mathrm{P,exp}}(\omega)/S_{\mathrm{P,cal}}(\omega) = [\Delta V_{\mathrm{exp}}(\omega)/\Delta V_{\mathrm{cal}}(\omega)]e^{i[\varphi_{\mathrm{exp}}(\omega)-\varphi_{\mathrm{cal}}(\omega)]}$, which is the ratio of the frequency-domain experimental and calculated electro-optic signals in our reference experiment on the THz emission from the ZnTe crystal. The amplitude and phase of this non-ideality factor, shown in Fig. 6b, are rather small, confirming our correct understanding of the THz propagation conditions in our experiment. The small deviations between the experiment and first-principles-based calculation at lowest and highest frequencies can be caused by the possible slight off-axis propagation of the THz beam in our spectrometer, and the assumption of the pencil electro-optic sampling gating beam in the calculation, instead of the Gaussian beam of the same effective area, used in the experiment. We however emphasize here, that the reconstruction of the magnetization dynamics $M(t)$ only relied on the experimentally determined spectrometer function $f_{\mathrm{prop}}(\omega)f_{\mathrm{d}}(\omega)$, and the full numerical modeling of our spectrometer was performed for control purposes.

It should be noted here, that for the accurate signal reconstruction the bandwidth of the detector should exceed that of the emitter. This is demonstrated in Fig. 5b: the bandwidth of the electric dipole emission from the reference emitter, 0.5 mm ZnTe crystal, does indeed exceed that of MgO/Fe/MgO and MgO/Fe/Pd samples. We remind that all the THz signals in this work were detected in one and the same 1 mm ZnTe crystal. This indeed allows for the accurate reconstruction of the quantities of interest – the sources $P(t)$ and $M(t)$.

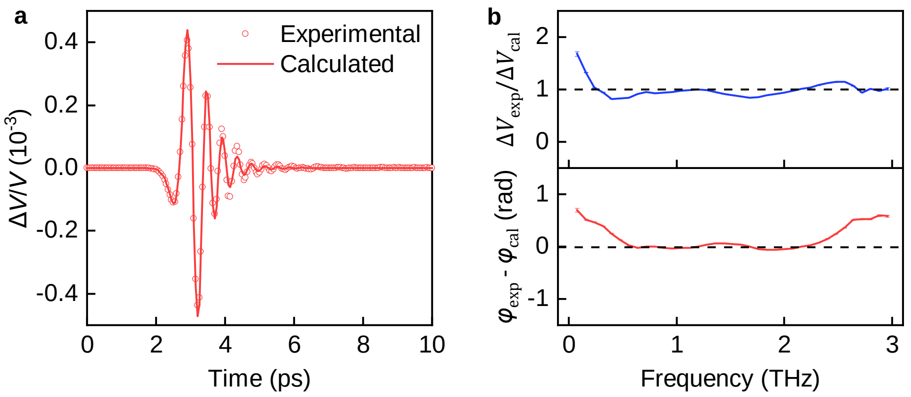

Supplementary Figure 6. Comparison of experimental and calculated electro-optic signals. **a**, The experimental and calculated electro-optic signals of THz radiation emitted from the reference emitter, <110> ZnTe crystal of 0.5 mm thickness. **b**, The amplitude and the phase of the non-ideality factor in the numerical modeling of the complete THz propagation in our experiment, demonstrating high numerical accuracy of our signal recovery approach. The error bars in b are the standard error calculated from multiple measurements.

Supplementary Note 5: Sample preparation

The thicknesses were confirmed by small-angle X-ray diffraction for these samples by means of a Rigaku SmartLab® X-ray diffractometer equipped with a monochromatic source (Ge(220)×2) delivering a Cu Kα_1 incident beam (45 kV, 200 mA, $\lambda = 0.154056$ nm), see Fig. 7a). Using the same equipment, high-angle X-ray diffraction shows that Fe crystallizes well in the classical bcc lattice structure (Fig. 7b).

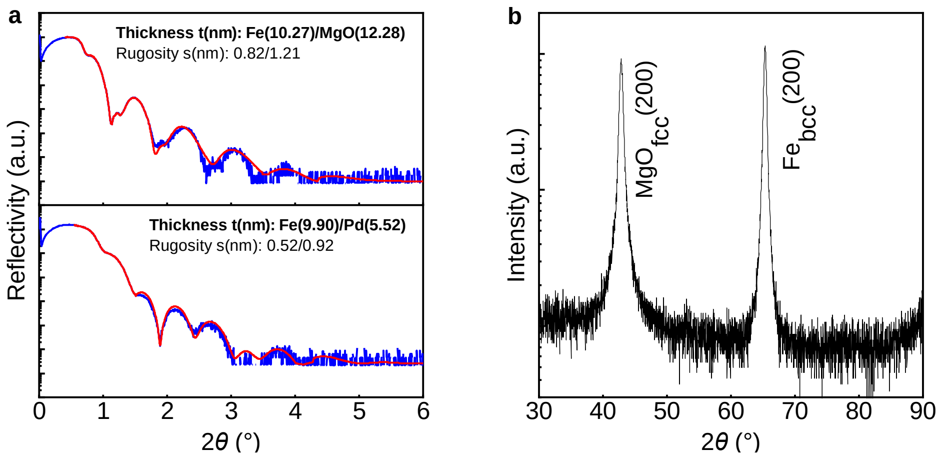

Supplementary Figure 7. The thicknesses of Fe/MgO and Fe/Pd and the lattice structure of Fe crystal. **a**, The thickness and rugosity of Fe/MgO and Fe/Pd measured with small-angle X-ray diffraction and the data fitting. **b**, Lattice structure of Fe/MgO measured with high-angle X-ray diffraction.

Supplementary References

1. E. Beaurepaire, G. M. Turner, S. M. Harrel, M. C. Beard, J. Y. Bigot, and C. A. Schmuttenmaer, "Coherent terahertz emission from ferromagnetic films excited by femtosecond laser pulses," Appl. Phys. Lett. **84**, 3465–3467 (2004).
2. M. Battiato, K. Carva, and P. M. Oppeneer, "Superdiffusive Spin Transport as a Mechanism of Ultrafast Demagnetization," Phys. Rev. Lett. **105**, 027203 (2010).
3. T. Kampfrath, M. Battiato, P. Maldonado, G. Eilers, J. Nötzold, S. Mährlein, V. Zbarsky, F. Freimuth, Y. Mokrousov, S. Blügel, M. Wolf, I. Radu, P. M. Oppeneer, and M. Münzenberg, "Terahertz spin current pulses controlled by magnetic heterostructures," Nat. Nanotechnol. **8**, 256–260 (2013).
4. J. Faure, J. Van Tilborg, R. A. Kaindl, and W. P. Leemans, "Modelling laser-based table-top THz sources: Optical rectification, propagation and electro-optic sampling," Opt. Quantum Electron. **36**, 681–697 (2004).
